# Impact of a tenofovir disoproxil fumarate plus ritonavir-boosted protease inhibitor-based regimen on renal function in HIV-infected individuals: a prospective, multicenter study

**DOI:** 10.1186/1471-2334-13-301

**Published:** 2013-07-01

**Authors:** Ying Cao, Yang Han, Jing Xie, Qu Cui, Lixia Zhang, Yijia Li, Yanling Li, Xiaojing Song, Ting Zhu, Taisheng Li

**Affiliations:** 1 Department of Infection, Peking Union Medical College Hospital, Chinese Academy of Medical Sciences and Peking Union Medical College, Beijing 100730, China

**Keywords:** Antiretroviral Therapy, HIV, Renal Function, Tenofovir, Protease Inhibitor

## Abstract

**Background:**

The aim of this study was to investigate the impact of a tenofovir disoproxil fumarate (TDF) plus ritonavir-boosted protease inhibitor (PI/r) regimen on renal function in Chinese HIV-infected patients.

**Methods:**

Seventy-five HIV-1 infected patients failing first-line antiretroviral therapy (ART) comprised the TDF+PI/r group. Seventy-five HIV-1 infected patients matched for gender, age, and renal function made up the control. All subjects completed follow-up visits over 48 weeks. CD4 cell count, plasma HIV-1 viral load, and urine protein level were assessed at the trial start (baseline, week 0) and at week 48. The serum creatinine and estimated glomerular filtration rate (eGFR) were monitored at each follow-up point. Change in eGFR from baseline to week 48 was also compared.

**Results:**

Compared to control, the TDF+PI/r group exhibited higher levels of serum creatinine (79 vs. 69.7 μmol/L, *P*<0.001) and a lower rate of eGFR (93.0 vs. 101.6 ml/min/1.73m^2^, *P*=0.009) at the end of week 48. Patients treated with TDF+PI/r showed greater decline in eGFR than control (−8.8 vs. 6.4ml/min/1.73m^2^, *P*<0.001). Compared to baseline renal function of the control group, the TDF+PI/r group exhibited a greater median decline in eGFR at the end of week 48 (*P*<0.001).

**Conclusions:**

We found that a TDF+PI/r based ART regimen resulted in greater renal function decline over 48 weeks. Therefore, renal function should be monitored especially when TDF is used in combination with PI/r.

**Trial registration:**

ClinicalTrials.gov identifier: NCT00872417

## Background

Combined antiretroviral therapy (cART) has reduced the mortality and morbidity associated with human immunodeficiency virus type 1 (HIV-1) infection and the risk of HIV-infected patients’ progression to Acquired Immune Deficiency Syndrome (AIDS). Side effects have been reported in the past decades. Complications related to long-standing HIV infection and treatment, such as the nephrotoxic effects, have become the focus of researchers. cART can itself cause renal toxic effects directly by inducing acute interstitial nephritis, crystal nephropathy, and renal tubular disorders [1]. cART can also contribute to renal dysfunction indirectly via drug interactions. Kidney damaged induced by the nucleotide analogue reverse transcriptase inhibitor (NRTI) tenofovir is a well studied drug that causes kidney damage in HIV patients. Tenofovir was licensed for the treatment of HIV-infected patients in the USA in 2001 [2], is well tolerated, and has no evidence of long-term mitochondrial toxicity effects. Tenofovir disoproxil fumarate (TDF) is recommended as a first-line therapy in HIV treatment by both the American Academy of HIV Medicine [3] and the European AIDS Clinical Society [4].

TDF is eliminated largely by glomerular filtration, and 20%-30% by active renal proximal tubular secretion [5]. Previous case reports reported TDF-associated renal toxicity, including acute tubular necrosis [6], Fanconi syndrome [7-9], and ultimately, renal failure [10] characterized by a decline in glomerular filtration rates (GFRs) and hypophosphatemia [11,12]. Studies with primarily Caucasian patients report low rates of tenofovir-associated nephrotoxicity, and severe renal impairment is rare in clinical trials of tenofovir-containing first line antiretroviral therapy (ART) regimens [13,14]. Recently, Alana Brennan et al. reported that 2.4% South African (predominately black) HIV patients receiving a tenofovir-containing regimen experienced nephrotoxicity, and 7.8% died during 48 months of follow-up [15].

Little is known about renal safety of TDF in Asian patients on tenofovir. TDF has been provided at no cost by the Chinese government as a second-line therapy to HIV-infected individuals who failed first-line treatment since mid 2009. Moreover, the government has classified TDF as a first-line therapeutic since 2012. It is thus urgently necessary to determine TDF effects on renal function. In this study, we report the outcome of a 48-week prospective study on renal safety data of TDF plus ritonavir-boosted protease inhibitor (PI/r) based cART regimens in Chinese adult HIV-infected patients.

## Methods

### Patients

This was a prospective, observational study conducted at eight research centers in China. Patients were recruited from several different regions of the country including Beijing, Shanghai, Guangzhou, Shenzhen, Fuzhou, Henan, and Yunnan provinces between November 2008 and January 2010. Seventy-five HIV-1 infected patients with first-line cART regimen treatment failure were enrolled in the TDF+ ritonavir-boosted protease inhibitor (PI/r) group. Patients all received second-line cart: TDF + ritonavir-boosted lopinavir (LPV/r) + Lamivudine (3TC). Inclusion criteria of this group included: (1) 18–65 years of age , (2) first-line cART failure defined as receiving first-line cART (e.g., Thirty five patients treated with Zidovudine (AZT) + 3TC + Nevirapine (NVP) / Efavirenz (EFV), twenty patients treated with Stavudine (D4T) + 3TC + NVP / EFV, fifteen patients treated with AZT + Didanosine (DDI) + NVP / EFV and five patients treated with D4T + DDI + NVP / EFV.) for one year with plasma HIV-1 viral load (VL) more than 1000 copies per milliliter (cps/ml), and (3) available estimated glomerular filtration rate (eGFR). The main exclusion criteria were (1) patients who had received initial cART regimes containing TDF and/or protease inhibitors (PIs) (2) serum creatinine >1.5 times the upper limit of normal, (3) eGFR< 50ml/min/1.73m^2^, and (4) anticipated poor adherence. Seventy-five HIV-1 infected cART-naïve patients matched for gender, age, serum creatinine and eGFR were selected from a multicenter cohort study population and made up the control group. Control patients were given a cART regimen of D4T/AZT+NVP+3TC. All subjects complied to follow-up visits over 48 weeks from 2008 to 2010. To further investigate the influence of TDF+PI/r-based cART regimens on renal function between individuals with normal and abnormal renal function, the TDF+PI/r group was divided into a normal renal function (eGFR≥90 ml/min/1.73m^2^) sector and an abnormal one (90>eGFR≥50 ml/min/1.73m^2^) based on eGFR baseline. To note, the control group subjects were not on an ART regimen, but TDF+PI/r patients were receiving a first line ART regimen before the start, switching to a second line ART regimen (TDF + 3TC+ LPV/r) at the study’s beginning. Informed consent was obtained from each participant. Ethics approval was obtained from the Institutional Review Board of Peking Union Medical College Hospital (PUMCH).

### Measurements

The following clinical and laboratory data were collected and recorded on case report forms: age, gender, race, duration of HIV seropositive, weight, height, hypertension, diabetes mellitus, smoking, CD4+ cell count, plasma HIV-1 viral load, urine protein level, serum creatinine, and eGFR. Hypertension was defined as either blood pressure greater than 140/90mm Hg on at least two of any three preliminary clinic visits, or the use of an antihypertensive medication. Diabetes mellitus was defined by physician diagnosis or by patient use of an antidiabetic agent. CD4 cell count, plasma HIV-1 viral load, and urine protein level were monitored at start and week 48. Serum creatinine and eGFR were monitored at start and at weeks 0, 4, 8, 12, 24, 36, and 48. Change in eGFR from week 0 to 48 for TDF+PI/r and control groups was compared. Urine protein level and serum creatinine were administered by PUMCH clinical laboratory departments. The eGFR was calculated using the four-variable Modification of Diet in Renal Disease (MDRD) [16]. Urine protein was measured by a urine dipstick. Proteinuria was defined as ≥1+ on a urine dipstick. The CD4+ cell count was detected from Ethylenediaminetetraacetic acid (EDTA)-anticoagulated whole blood by FACS-Calibur (BD Biosciences, NJ, USA) using standard techniques according to the manufacturer’s protocol. Separated plasma was immediately frozen at −80°C and transported to PUMC Hospital’s central laboratory for viral load testing using the COBAS Ampliprep/TaqMan 48 real-time reverse transcription PCR (RT-PCR) (Roche, CA, USA) according to manufacturer’s instructions. The assay working range was between 40 and 1,000,000 cps/ml.

### Statistical analysis

All analyses were performed on the intent-to-treat (ITT) population. Missing values were imputed for the analysis with the last-observation-carried-forward method. Continuous variables were assessed with the Student’s *t* test or Mann–Whitney test appropriately. Categorical variables were assessed with the Chi-square test. Differences in changes in serum creatninine and eGFR at 48 weeks were assessed using the Wilcoxon rank sum test to compare median values of continuous data. Absolute differences for each group in serum creatinine and eGFR were respectively analyzed during the follow-up using repeated measures of variance. The Wilcoxon signed rank test was used to assess trends in serum creatinine or eGFR during the follow-up. For all tests, a *P*-value less than 0.05 was considered statistically significant. Statistical analysis was conducted via SPSS version 16.0 (SPSS Inc., Chicago, IL, USA).

## Results

A summary of patient characteristics in both TDF+PI/r and control groups are summarized in Tables 1 and 2. Patients were well matched, with no wide demographic variation between cohorts. Further, no significant differences in hypertension, diabetes, smoking, body mass index (BMI), CD4+ cell count, plasma HIV-1 viral load, and urine protein level were detected (Table 1). The duration of HIV seropositive was significantly longer in TDF+PI/r patients compared to control (median [inter-quartile range, IQR], 65[26–139] vs. 22[19–34] months, *P*<0.001).

**Table 1:** **Characteristics of patients in TDF+PI/r and control groups**^**a**^

**Variable**	**TDF+PI/r group (n=75)**	**Control group (n=75)**	***P***
**Male**	58(77.3)	53(70.7)	0.352
**Age (years)**	44 (41–50)	42 (34–53)	0.322
**Race**			0.363
Han	74 (98.7)	71 (94.7)	
Other	1 (1.3)	4 (5.3)	
**Duration of HIV seropositive (months)**	65 (26–139)	22 (19–28)	<0.001
**Smoking**			
Yes	32(42.7)	27(36.0)	0.403
No	43(57.3)	48(64.0)	
**Hypertension**			0.347
Yes	7(9.3)	4(5.3)	
No	68(90.7)	71(94.7)	
**Diabetes**			0.332
Yes	4(5.3)	1(1.3)	
No	67(89.3)	68(90.7)	
Unknown	4(5.3)	6(8.0)	
**BMI**^**b**^**(kg/m**^**2**^**)**	21.6 (20.1-23.5)	20.6 (19.5-23.0)	0.129
**CD4 cell count (cells/μl)**	157(63–267)	185 (104–259)	0.509
**Viral load (log10 copies/ml)**	4.4 (3.7-4.8)	4.5 (3.9-4.9)	0.117
**Proteinuria**			0.631
Positive	11(14.7)	9 (12)	
Negative	64(85.3)	66 (88)	
**Serum creatinine (μmol/L)**	71(65–81)	71.4(62–81.7)	0.263
**eGFR**^**c**^**(ml/min/1.73 m**^**2**^**)**	101.6 (85.6-117.2)	105.1 (76.8-110.3)	0.376

Note.

^a^Values are expressed as median (interquartile range) or number (percentage).

^b^BMI =weight (kg)/(height (m))^2^.

^c^eGFR = 186 × (Serum creatinine (mg/dL))^-1.154^ × (age(years))^-0.203^ × (0.742 if female).

**Table 2:** **Characteristics of patients in TDF+PI/r and control groups at the end of 48 weeeks**^**a**^

**Variable**	**TDF+PI/r group (n=75)**	**Control group (n=75)**	***P***
**BMI**^**b**^**(kg/m**^**2**^**)**	21.9 (20.1-23.7)	21.0 (19.3-22.9)	0.083
**CD4 cell count (cells/μl)**	279 (196–380.5)	292.0 (207.0-394.0)	0.736
**Viral load (log**_**10**_**copies/ml)**	1.6 (1.6-1.8)	1.6 (1.6-1.7)	0.064
**Proteinuria**			0.071
Positive	9 (12)	3(4)	
Negative	66 (88)	72 (96)	
**Serum creatinine (μmol/L)**	79 (70–85)	69.7 (62–80.3)	<0.001
**eGFR**^**c**^**(ml/min/1.73 m**^**2**^**)**	93.0 (82.4-104.0)	101.6 (86.8-121.6)	0.009
**Change in eGFR (ml/min/1.73 m**^**2**^**)**	−8.8(−18.5-3.3)	6.4(−3.8-16.5)	<0.001

Note.

^a^Values are expressed as median (interquartile range, IQR) or number (percentage).

^b^BMI =weight (kg)/(height (m))^2^.

^c^eGFR = 186 × (Serum creatinine (mg/dL))^-1.154^ × (age(years))^-0.203^ × (0.742 if female).

Using intent-to-treat (ITT) analysis at the end of week 48, TDF+PI/r patients exhibited higher levels of serum creatinine (median[IQR], 79.0[70.0-85.0] vs. 69.7[62–80.3] μmol/L, *P*<0.001) and a significantly lower rate of eGFR (median[IQR], 93.0[82.4-104.0] vs. 101.6[86.8-121.6] ml/min/1.73m^2^, *P*=0.009). BMI was similar for TDF+PI/r and control groups (median[IQR], 21.9[20.1-23.7] vs. 21.0[19.3-22.9] kg/m^2^, *P*=0.083). No difference was noted in CD4 cell count (median[IQR], 279[196–380.5] vs. 292.0[207.0-394.0] cells/μl, *P*=0.736) or HIV-1 RNA viral load (median[IQR], 1.6[1.6-1.8] vs. 1.6[1.6-1.7] log_10_ copies/ml, *P*=0.064). Importantly, however, median eGFR did change from start to 48 weeks for TDF+PI/r patients vs. control (median[IQR], -8.8[−18.5-3.3] vs. 6.4[−3.8-16.5] ml/min/1.73m^2^, *P*<0.001). Patients treated with TDF+PI/r showed declining eGFR levels.

**Figure 1:**
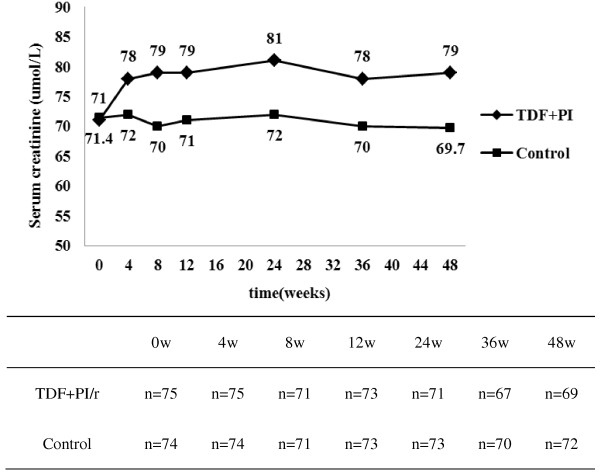
Changes in median serum creatinine concentrations at 0, 4, 8, 12, 24, 36, and 48 weeks in TDF+PI/r and control groups.

The changes in serum creatinine concentrations during the 48 week follow-up period are shown in Figure 1. For control, median serum creatinine concentrations varied between 69.7 and 72.0 ml/min/1.73m^2^ for baseline and the 48-week point, respectively, and there existed no significant differences at each follow-up point in between (*P*=0.638). For the TDF+PI/r group, the median [IQR] serum creatinine concentration was 71.0 [65.0-81.0] μmol/L at baseline, and varied between 78.0 and 81.0 μmol/L between 4 and 48 weeks. We found that compared with baseline, the median values of serum creatinine for the 4-week point were significantly higher in TDF+PI/r group (median [IQR], 71.0 [65.0-81.0] vs.78.0 [70.0-85.0] μmol/L) (*P*=0.002). However, the median values of serum creatinine between the 4-week point and the 48-week point were no significant differences in TDF+PI/r group (median [IQR], 78.0 [70.0-85.5] vs.79.0 [70.0-85.0] μmol/L) (*P*=0.719). So it demonstrated that the values of serum creatinine increased significantly initially (0–4 weeks), and then (5-48weeks) maintained at the stable level in TDF+PI/r group.

**Figure 2:**
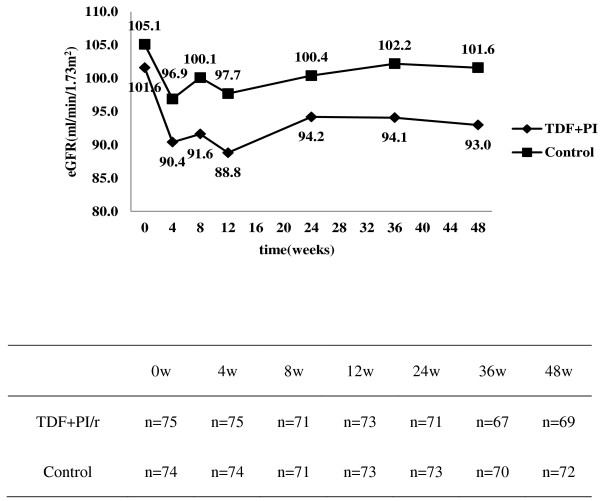
Changes in median eGFR at 0, 4, 8, 12, 24, 36, and 48 weeks in TDF+PI/r and control groups.

Changes in eGFR during the 48 week follow-up are shown in Figure 2. For control, median eGFR varied between 96.9 and 105.1 ml/min/1.73m^2^ with no significant differences at each follow-up point. For the TDF+PI/r group, median [IQR] starting eGFR was 101.6[85.6-117.2] ml/min/1.73m^2^ and varied between 88.8 and 94.2 ml/min/1.73m^2^ between 4 and 48 weeks. Compared with baseline, the median eGFR for the 4-week point were significantly lower in TDF+PI/r group (median [IQR], 101.6[85.6-117.2] vs. 90.4[82.8-103.4] ml/min/1.73m^2^) (*P*=0.001). However, the median values of eGFR between the 4-week point and the 48-week point were no significant differences in TDF+PI/r group (median [IQR], 90.4[82.8-103.4] vs. 93.0[82.4-104.0] ml/min/1.73m^2^) (*P*=0.725). So it showed that the median values of eGFR decreased significantly initially (0–4 weeks), and within 5–48 weeks, median eGFR remained relatively stable in participants treated with TDF + LPV/r + 3TC. We also calculated creatinine clearance (CrCl) using the Cockcroft–Gault equation, and saw a similar trend, as eGFR changes throughout the 48 weeks between the two groups (data not shown).

**Figure 3:**
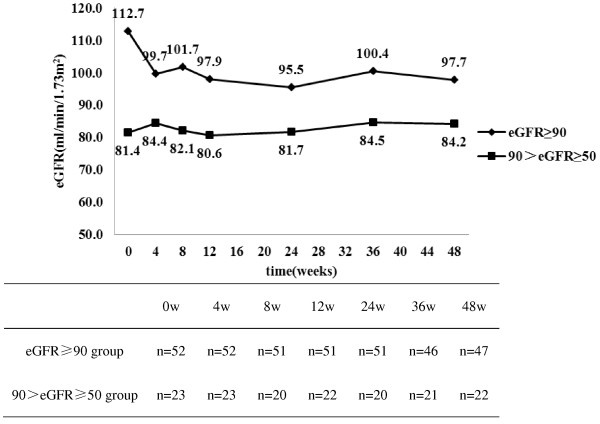
**Changes in median eGFR over time in the TDF+PI/r group, by eGFR normal (eGFR≥90 ml/min/1.73m**^**2**^**) and abnormal (90>eGFR≥50 ml/min/1.73m**^**2**^**) baselines.**

To further analyze the impact of TDF+PI/r-based cART regimens on renal function between renal function normal and abnormal patients, 75 TDF+PI/r patients were stratified by kidney function into normal (eGFR≥90 ml/min/1.73m^2^) or abnormal (90>eGFR≥50 ml/min/1.73m^2^) groups: 52 and 23 patients were respectively divided into these groups. Significant differences in eGFR levels between these groups at baseline (median[IQR], 112.7[100.9-121.3] vs. 81.4[76.9-85.4], *P*<0.001) and at week 48 (median[IQR], 97.7 [86.9-110.3] vs. 84.2[70.6-94.5], *P*<0.001) were observed. Changes in median eGFR during 48 weeks of TDF+PI/r-based cART treatment between the two groups are shown in Figure 3. As Figure 3 highlights, baseline eGFR normal patients showed greater changes in median eGFR than baseline eGFR abnormal patients at week 4, 8, 12, 24, 36, and 48 (*P*<0.001), but renal function always fluctuated within normal range. For eGFR abnormal patients, no significant differences in median eGFR were found at 0, 4, 8, 12, 24, 36, and 48 weeks. For these patients, magnitude of eGFR decline was mild, and severe renal dysfunction was not observed during follow-up. Baseline eGFR normal patients exhibited greater decline in eGFR than eGFR abnormal patients at 4, 8, 12, 24, 36, and 48 weeks, but renal function still fluctuated within the normal range. For two (8.7%) patients, eGFR decreased to less than 50ml/min/1.73m^2^, but severe renal dysfunction was not detected during the 48 week follow-up. After 48 weeks, no significant difference in proteinuria incidence between the two groups (12% vs. 4%, *P*=0.071) was apparent.

## Discussion and conclusions

Here, we present novel data on the influence of TDF combination with PI/r cART regimens on renal function in Chinese adult HIV-infected patients over a 48 week period. We found that patients exposed to TDF + LPV/r + 3TC exhibited higher levels of serum creatinine, a lower eGFR rate, and greater renal function decline than control. Importantly, our data demonstrated that serum creatinine rapidly increased, while eGFR remarkably declined during the first 4 weeks.

Only a few patients can afford the cost of TDF if TDF was used as first-line therapy drug. Patients who failed first-line treatment could receive cART containing TDF because TDF was a free second-line therapy provided by the Chinese government to HIV-infected individuals who failed first-line treatment since mid-2009. Patients in the TDF group were infected with HIV for longer than control, and were previously exposed to ART agents. Although patients in the TDF group were HIV seropositive longer, they received first-line cART (e.g., AZT + 3TC +NVP/EFV, D4T + 3TC + NVP/EFV, AZT + DDI + NVP/EFV or D4T + DDI + NVP/EFV) for over a year. To our knowledge, first-line therapies mentioned previously were not known to be associated with renal damage. However, using cART and suppressing HIV RNA may improve kidney function [17], reduce the rate of eGFR decrease [18], and/or reduce the risk of renal events [19]. Patients who received initial cART regimes containing TDF and/or PIs were excluded from our study, and baseline serum creatinine, eGFR and HIV-1 RNA viral load were not significantly different between TDF+PI/r and control groups.

Nancy Crum-Cianflone et al. [20] evaluated the impact of tenofovir and a boosted PI on renal dysfunction among 150 HIV-infected Caucasian patients Initiating both tenofovir and boosted PI therapy greatly impacted kidney function: 78 (52%) of subjects showed a reduction in eGFR, the median change being −12.1 ml/min/1.73m^2^ [95% confidence interval (CI), -9.1 to −14.1 ml/min/1.73m^2^]. Moreover, 30% lost >10 mL/min/1.73m^2^ during the 2-year follow-up period. Goicoechea et al. [21] compared the estimated decline in renal function among Western HIV-1 infected patients receiving either TDF+PI/r- (n=51) or non-TDF- containing (n=66) regimens. They demonstrated that TDF+PI/r patients showed a greater rate of decline in creatinine clearance (CrCl) than the non-TDF-containing group (for MDRD, -14.7 vs. -4.78 mL/min/1.73m^2^/year). Consistent with observations in the Western population, median changes of eGFR were −8.8 ml/min/1.73m^2^ in TDF+PI/r and 6.4 ml/min/1.73m^2^ in control. Patients treated with TDF and PI/r showed greater decline in renal function over 48 weeks compared to non-TDF-containing regimens. Gallant JE et al. also illustrated that patients taking TDF and a PI/r exhibited a greater median decline in eGFR than those taking TDF and a non-NRTI at 6 months, with trends at 12 and 24 months [12]. Data from the HIV Outpatient Study (HOPS) cohort, however, showed no differences in rate of creatinine clearance at 12 months between patients who were treated with either tenofovir, non-NRTIs, or ritonavir-boosted protease inhibitors (atazanavir or lopinavir) [22]. A smaller renal function change in PI/r-treated patients may result from differences in patient characteristics. Compared with the HOPS cohort, only antiretroviral-experienced patients age 18–65 with eGFR more than 50 ml/min/1.73m^2^ and less advanced HIV disease were included in our study. Moreover, the comparator group contained both TDF- and other PI+TDF-treated patients. This may have influenced the decreased renal function observed in their study, reducing the window to discern an effect between treatment groups.

For tenofovir-induced nephrotoxic effects, approximately 70% are observed with concomitant use of low-dose ritonavir [1]. Due to a PI/r and tenofovir interaction, renal clearance of tenofovir is retarded. Kiser JJ et al. observed that patients taking tenofovir + LPV/r showed a 17.5% lower tenofovir renal clearance than those taking TDF alone, even after adjusting for GFR differences [23]. The plasma concentration of tenofovir can be increased by approximately 20-37% in PI/r-containing regimens [24,25]. Studies have also highlighted that ritonavir is a potent inhibitor of multidrug resistance-associated protein 2 (MRP-2) [26]. Postulated mechanisms include a potential unidentified cofactor normally excreted by MRP2 potentiating TDF toxicity or competing for TDF excretion at MRP4 [27]. Whether this mechanism is due to PI/r competitively inhibiting renal tubular transporter function, decreasing TDF renal excretion needs further elucidation.

For our analyses of kidney function over time among patients using TDF+LPV/r+3TC, the impact of TDF+ PI/r was apparent within the first 4 weeks and stabilized to 48 weeks. Consistent with an observation from a Development of Antiretroviral Therapy (DART) trial in a large cohort of HIV-infected African adults from Uganda and Zimbabwe, changes in eGFR were also observed predominantly during early exposure to TDF cART regimens, with kidney function stabilization after ~4 weeks [28]. Contrastingly, in the EuroSIDA (AIDS across Europe) study, data from larger European cohorts have demonstrated an association between TDF cumulative exposure and renal toxicity increases [29]. Andrew N. Phillips et al. performed a prospective study comprised of 85% white patients. 89.8% of enrolled participants were cART-experienced patients, and 99.7% of patients were already receiving a TDF-containing cART regimen. Many enrolled participants exhibited preexisting risk factors for CKD. In the TDF group, 21.7% patients were using nephrotoxic drugs, such as pentamidine, cidofovir, acyclovir, foscarnet, or amphotericin B at or before the trial’s start.

In our study, patients were Chinese, and 90% were of the Han race. All patients in TDF+PI/r group had never taken TDF and/or PIs-containing cART regimens, nor did they use nephrotoxic drugs prior to the trial. Thus, a plausible explanation for this discrepancy resides in the longer follow-up of the EuroSIDA cohort, genetics, difference in treatment history, and inclusion patients who may have had pre-existing risk factors for CKD, subsequently increasing the risk of TDF-related CKD.

The impact of a TDF+PI/r-based cART regimen on renal function was analyzed in patients stratified by baseline eGFR. Baseline eGFR was divided into two categories (eGFR≥90 and 90>eGFR≥50 ml/min/1.73m^2^). We expect an observable significant decline in patients’ renal function whose baseline is 90>eGFR≥50 ml/min/1.73m^2^. We were thus surprised to detect no significant differences in median eGFR at 0, 4, 8, 12, 24, 36 and 48 weeks for patients with baseline 90>eGFR≥50 ml/min/1.73m^2^. Perhaps patients with this basal eGFR remain insensitive to nephrotoxic side effects, but may be hypersensitive to renal dysfuntion. Our cutoff for group sorting may be too broad to detect eGFR changes, since TDF impact on GFR is normally small. Studies confirming our hypotheses require further elucidation.

Early detection and diagnosis of renal dysfunction are essential for preventing or at least slowing kidney function decline to ultimately improve outcome in HIV-infected patients. Individuals with underlying impaired kidney function and patients whose kidney damage was drug-induced would likely benefit from vigilant renal follow-up. Particularly, patients receiving tenofovir may benefit from frequent assessments of kidney function, serum phosphate levels, and urinalyses to monitor for early signs of nephrotoxicity [6,30]. The HIV Medicine Association of the Infectious Diseases Society of America (IDSA) recommends that patients receiving TDF meet any of three criteria: eGFR<90 mL/min/1.73 m^2^, use of medications eliminated through renal secretion, comorbid diseases following a ritonavir-boosted protease inhibitor regimen, should have kidney function (via eGFR) and serum phosphate measured no less frequently than every 6 months, and also should be analyzed for proteinuria and glycosuria [31]. Evidence is lacking in instructing patients about frequency of assessment. In agreement with the IDSA, our results suggest that eGFR should be monitored over the first 4 weeks of follow-up when TDF is combined with PI/r. Patients who exhibited decrease eGFR with TDF+PI/r probably are candidates for more frequent assessment of kidney function, serum phosphate levels, and urinalyses, and additionally have their kidney function evaluated for a longer term. TDF+PI/r-based cART regimens should be carefully considered in patients with decreased kidney function.

Our study reports that incidences of proteinuria were not significant for both groups at weeks 0 or 48. Tenofovir is thought to primarily affect proximal renal tubular function [32], yet glomerular function may be lightly affected, so proteinuria incidences were not noticeable in urine.

Our study does have limitations. First, patients in the TDF+ PI/r group were receiving first-line cart prior to the study, while control patients were naïve. Second, only 3 patients older than 60 were enrolled. This study was thus not designed to decipher an effect of TDF on renal function of older patients, and the safety of TDF on older patients should be assessed in the future. Third, patients whose serum creatinine > 1.5 times the upper limit of laboratory normal and/or eGFR< 50ml/min/1.73m^2^ were excluded from our study. The influence of TDF based cART regimens on renal function in HIV-1 with compromised renal function remains poorly understood. Patients with renal dysfunction treated with TDF cART regimens should be frequently monitored by nephrologists. Finally, our last time point was 48 weeks. Further follow-up in a larger cohort would be required to more accurately determine long-term TDF-related nephrotoxicity. In view of these limitations, a prospective long-term multicenter clinical trial is being considered to investigate the potential impact of TDF on renal function in Chinese HIV-infected individuals. Future trials may include older patients and patients with moderate to severe renal dysfunction.

In conclusion, TDF+PI/r based cART regimens were associated with a higher level of serum creatinine and a greater decline in renal function over 48 weeks compared to control. For patients with baseline eGFR ≥90 ml/min/1.73m^2^, TDF+PI/r based cART regimens could influence non-progressive eGFR decreases, but the impact of TDF+PI/r based cART regimens on eGFR in patients with abnormal renal function seemed to be relatively minor. Additionally, renal function rapidly decreased during the first four weeks. Our results suggest that renal function should be monitored over the first four weeks of follow-up, especially when TDF is combined with PI/r.

## Competing interest

The authors declared that they have no competing interest.

## Authors’ contributions

YC assisted with the study design, performed the data statistical analyses, and wrote this manuscript. YH recruited patients and performed HIV-1 viral load tests. JX determined CD4+T cell count. QC and YL assisted in drafting the manuscript. LZ performed the renal function assay. YL and XS collected clinical data. TZ performed the HIV-1 viral load test. TL designed and supervised this study. All authors read and approved the final manuscript.

## Pre-publication history

The pre-publication history for this paper can be accessed here:

http://www.biomedcentral.com/1471-2334/13/301/prepub
